# Review of control strategies for robotic movement training after neurologic injury

**DOI:** 10.1186/1743-0003-6-20

**Published:** 2009-06-16

**Authors:** Laura Marchal-Crespo, David J Reinkensmeyer

**Affiliations:** 1 Department of Mechanical and Aerospace Engineering, University of California, Irvine, USA; 2 Department of Biomedical Engineering, University of California, Irvine, USA

## Abstract

There is increasing interest in using robotic devices to assist in movement training following neurologic injuries such as stroke and spinal cord injury. This paper reviews control strategies for robotic therapy devices. Several categories of strategies have been proposed, including, assistive, challenge-based, haptic simulation, and coaching. The greatest amount of work has been done on developing assistive strategies, and thus the majority of this review summarizes techniques for implementing assistive strategies, including impedance-, counterbalance-, and EMG- based controllers, as well as adaptive controllers that modify control parameters based on ongoing participant performance. Clinical evidence regarding the relative effectiveness of different types of robotic therapy controllers is limited, but there is initial evidence that some control strategies are more effective than others. It is also now apparent there may be mechanisms by which some robotic control approaches might actually decrease the recovery possible with comparable, non-robotic forms of training. In future research, there is a need for head-to-head comparison of control algorithms in randomized, controlled clinical trials, and for improved models of human motor recovery to provide a more rational framework for designing robotic therapy control strategies.

## Introduction

**Figure 1:**
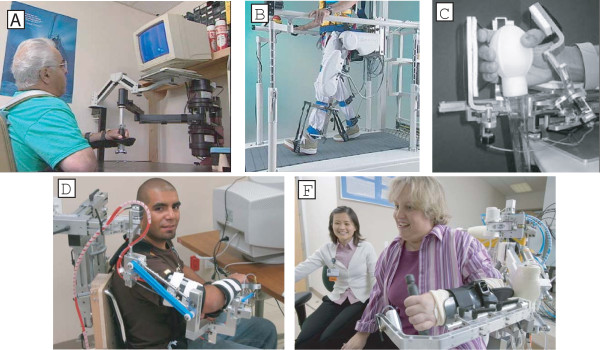
**Examples of robotic therapy devices using different types of assistance-based control algorithms**. Examples of robotic therapy devices using different types of assistance-based control algorithms. Two of the first devices to undergo clinical testing, MIT-MANUS and Lokomat, initially used proportional position feedback control to provide assistance. Newer software for MIT-MANUS [55] (A) adapts the timing and stiffness of the controller based on participant performance. New software for the Lokomat [10] (B) adjusts the shape of the desired stepping trajectory based on participant interaction forces, as well as the robot impedance. HWARD [157] (C), the hand robot, uses triggered assistance, which means that it allows free movement for a fixed time for each desired task, and then responds by moving the hand if the participant does not achieve the task. T-WREX [88] (D) uses passive gravity balancing to provide assistance, with the number of elastic bands determining the amount of assistance. Pneu-WREX [50] (F) builds a real-time computer model of the participant's weakness, and uses it to provide feedforward assistance with a compliant position controller.

**Figure 2:**
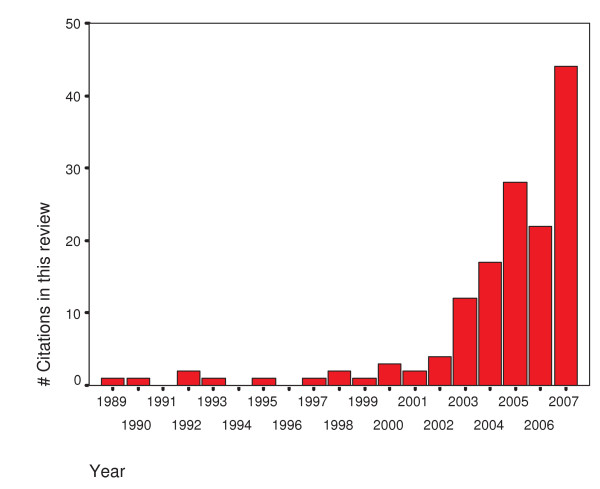
**Number of articles cited in this review article published each year for the last 20 years**. Number of articles cited in this review article published each year for the last 20 years. Note the exponential increase of publications in the last five years.

There is increasing interest in using robotic devices to help provide rehabilitation therapy following neurologic injuries such as stroke and spinal cord injury [1,2] (Figure 1). The general paradigm being explored [see Additional file 1] is to use a robotic device to physically interact with the participant's limbs during movement training, although there is also work that uses robots that do not physically contact the participant to "coach" the participant [3-5]. As can be seen in Figure 2, there was an exponential increase in papers in this field over the past ten years.

Much of this new work has focused on developing more sophisticated, many degrees-of-freedom robotic mechanisms, in order to support movement training of more complicated movements, such as walking [6-15], and multi-joint arm and hand movements [16-26]. Work has also focused on making devices portable so that they can be used during activities of daily living [11,27-31]. There has also been a progression in the development of control strategies that specify how these devices interact with participants. The purpose of this paper is to review this control strategy progression and to highlight some needed areas for future development.

The goal of robotic therapy control algorithms is to control robotic devices designed for rehabilitation exercise, so that the selected exercises to be performed by the participant provoke motor plasticity, and therefore improve motor recovery. Currently, however, there is not a solid scientific understanding of how this goal can best be achieved. Robotic therapy control algorithms have therefore been designed on an ad hoc basis, usually drawing on some concepts from the rehabilitation, neuroscience, and motor learning literature. In this review we briefly state these concepts, but do not review their neurophysiological evidence in any detail, focusing instead on how the control strategies seek to embody the general concepts.

One way to group current control algorithms is according to the strategy that they take to provoke plasticity: assisting, challenge-based, simulating normal tasks, and non-contact coaching [see Additional file 1]. Other strategies will likely be conceived in the future, but presently most algorithms seem to fall in these four categories, and we will use this categorization to organize this review.

The most developed paradigm is the assistive one. Assistive controllers help participants to move their weakened limbs in desired patterns during grasping, reaching, or walking, a strategy similar to "active assist" exercises performed by rehabilitation therapists. We will use the term "challenge-based" controllers to refer to controllers that are in some ways the opposite of assistive controllers because they make movement tasks more difficult or challenging. Examples include controllers that provide resistance to the participant's limb movements during exercise, require specific patterns of force generation, or increase the size of movement errors ("error amplification" strategies). The third paradigm, called haptic simulation, refers to the practice of activities of daily living (ADL) movements in a virtual environment. Haptic simulation has flexibility, convenience, and safety advantages compared to practice in a physical environment, as reviewed below. Finally, there is some work on robotic devices that do not physically contact the participant but instead serve as coaches, helping to direct the therapy program, motivate the participant, and promote motor learning. For such devices, it has been hypothesized that physically embodying the automated coaching mechanism has special merit for motivating participants [3]. Clearly, these strategies are not mutually independent, and in some cases multiple strategies could be combined and used in a complementary fashion. Further, assistance and challenge strategies can be viewed as different points on a continuum of either assistance or challenge; i.e. assistance is simply less challenge, and challenge is less assistance.

The goal of this paper is to review "high-level" rather than "low-level" robotic therapy control algorithms. By "high-level", we mean the aspects of the control algorithm that are explicitly designed to provoke motor plasticity. For many robots, such "high-level" algorithms are supported by low-level controllers that achieve the force, position, impedance, or admittance control necessary to implement the high-level algorithm. Research in robotic therapy devices has advanced the state-of-art in low-level force control also, for example, in control of pneumatic [21,22,27] and cable-based actuators [8,14,18,19,26,32-35], but these advances are not the focus of this article.

## Assistive controllers

Active assist exercise is the primary control paradigm that has been explored so far in robotic therapy development, and therefore the largest portion of this review is devoted to this topic. Active assist exercise uses external, physical assistance to aid participants in accomplishing intended movements. Physical and occupational therapists manually implement this technique in clinical rehabilitation on a regular basis, for both lower and upper extremity training.

Many rationales can be given for active assist exercise [see Additional file 1], none extensively verified in scientific studies. Active assist exercise interleaves effort by the participant with stretching of the muscles and connective tissue. Effort is thought to be essential for provoking motor plasticity [36,37], and stretching can help prevent stiffening of soft tissue and reduce spasticity, at least temporarily [38,39]. Another motivation is that by moving the limb in a manner that self-generated effort can not achieve, active assist exercise provides novel somatosensory stimulation that helps induce brain plasticity [40,41]. Another rationale is that physically demonstrating the desired pattern of a movement may help a participant learn to achieve the pattern [6,42]. Another rational, offered often in the context of locomotor training is that creating a normative pattern of sensory input will facilitate the motor system in reestablishing a normative pattern of motor output. Repetition of this normal pattern will reinforce it, improving unassisted motor performance [43,44]. Physically assisting movements can also help a participant to perform more movements in a shorter amount of time, potentially allowing more intense practice [45]. Another rationale, valid for tasks like walking or driving in which poor performance could lead to injury, is that assistance allows people to practice a task more intensively by making the task safe [28,46]. A related rationale is that assistance allows participants to progress in task difficulty, much as a young child learns to drive a bicycle with training wheels, starting with a tricycle and progressively reducing the support of the training wheels [6,46]. Finally, active assistance may have a psychological benefit. To quote a person post-stroke who participated in one of our studies "If I can't do it once, why do it a hundred times?" [47]. This quote emphasizes the fact that active assistance allows participants to achieve desired movements, and thus may serve to motivate repetitive, intensive practice by reconnecting "intention" to "action ".

On the other hand, there is also a history of motor control research that suggests that physically guiding a movement may actually decrease motor learning for some tasks (termed the "guidance hypothesis" [48], see review of guidance studies in motor learning in [46]). The reason is that physically assisting a movement changes the dynamics of the task so that the task learned is not the target task. Guiding the movement also reduces the burden on the learner's motor system to discover the principles necessary to perform the task successfully.

Guiding movement also appears in some cases to cause people to decrease physical effort during motor training. For example, persons with motor incomplete spinal cord injury who walked in a gait training robot that was controlled with a relatively stiff impedance-based assistive controller consumed 60% less energy than in traditional manually-assisted therapy [49]. Likewise, persons post-stroke who were assisted by an adaptively-controlled, compliant robot that had the potential to "take over" a reaching task for them decreased their own force output, letting the robot do more of the work of lifting their arm [50]. These findings suggest what might be termed the "Slacking Hypothesis": a robotic device could potentially decrease recovery if it encourages slacking; i.e. a decrease in motor output, effort, energy consumption, and/or attention during training.

Because providing too much assistance may have negative consequences for learning, a commonly stated goal in active assist exercise is to provide "assistance-as-needed", which means to assist the participant only as much as is needed to accomplish the task (sometimes termed "faded guidance" in motor learning research). Example strategies to encourage participant effort and self initiated movements include allowing some error variability around the desired movement using a deadband (an area around the trajectory in which no assistance is provided) triggering assistance only when the participant achieves a force or velocity threshold, making the robot compliant, or including a forgetting factor in the robotic assistance, as reviewed below.

After reviewing the literature, we decided to group active assistance control strategies into four conceptual categories [see Additional file 1]: impedance-based, counterbalance-based, EMG-based and performance-based adaptive assistance.

### Impedance-based assistance

The first assistive robotic therapy controllers proposed were proportional feedback position controllers [45,51-54]. Most subsequent robotic therapy devices, including devices for retraining upper extremity movement [17,18,20-22,24,25,34,45,55-70] and walking [8-12,15,28,31,71-76] have relied on a similar strategy of position feedback for providing assistance. More recent controllers have used more sophisticated forms of mechanical impedance than stiffness, including for example viscous force fields [71,77], creating virtual objects that assist in achieving the desired movement [78], or creating user-definable mechanical limits for complex postural or locomotor movements [28].

Assistive control strategies focus on a common, underlying idea: when the participant moves along a desired trajectory, the robot should not intervene, and if the participant deviates from the desired trajectory, the robot should create a restoring force, which is generated using an appropriately designed mechanical impedance. Controllers based on this principle provide a form of "assistance-as-needed", since assistance forces increase as the participant deviates from the desired trajectory. For example, for a proportional (plus derivative) position feedback controller, as the participant moves away from the desired trajectory, the controller force output increases proportionally, because the controller acts like a (damped) spring. Because humans show variability in their movements, a deadband is often introduced into impedance-based control schemes to allow normal variability without causing the robot to increase its assistance force [9,38,79]. Finally, these impedance-based assistance algorithms have been implemented in space only as defined above (e.g. a virtual channel that guides limb movement [9,17,18,56,80-82] or a region of acceptable pelvic motions during walking [28]) or in both time and space (e.g. a virtual channel with a moving wall [45,50,55,71]).

A variant of impedance-based assistance is triggered assistance, which allows the participant to attempt a movement without any robotic guidance, but initiates some form of (usually) impedance-based assistance after some performance variable reaches a threshold. This form of triggered assistance encourages participant self-initiated movement, which is thought to be essential for motor learning [36,37]. The sensed critical variable could be elapsed time [24,27,77,83,84], force generated by the participant [24,45,56,85], spatial tracking error [9,38,79], limb velocity [55,79,86], or muscle activity, measured with surface EMG [19,25,55,87]. For example, this triggering technique was used in initial studies with the ARM Guide [38,79] and MIT- MANUS robotic therapy devices [55,86], which assisted the participant in moving along a minimum jerk trajectory when the participant exceeded a movement error threshold, or moved faster than a velocity threshold, respectively. Similarly, in [79] the assistance is triggered when the participant is able to move faster than a performance-based velocity threshold. A force-based triggered assistance was initially applied with MIME robotic device [45], and more recently in [24,56,85]. In these studies the assistance is triggered when the participant pushes with a large enough force against the robotic device. Another approach consists in triggering assistance when the torque applied by the participant is below a threshold for a fixed time [77,83]. If the subject can not finish the task, the robot assists the participant to finish the task at a constant speed until the position error decreases below a threshold. Variations of time-triggered assistance have recently been used for the hand grasp robot HWARD [84], and reach and grasp robots Gentle/G [24] and RUPERT [27]. A danger of using triggered assistance is that a participant produces force or movement sufficient to activate the trigger, but then "rides" the robot, remaining ostensible passive for the rest of the movement.

### Counterbalancing assistance

Providing weight counterbalance to a limb is another assistance strategy that has been developed. Rehabilitation clinics have a long history of using devices to partially counterbalance the limbs, such as mobile arm supports, overhead slings, arm skateboards or towels that slide on tables, and harnesses for supporting body weight during walking. The use of swimming pools in rehabilitation can also be viewed as variant of this approach: active assistance is provided by virtue of the buoyancy of the body.

Recently developed devices implement passive counterbalancing schemes in a way that allows a greater range of motion than previous clinical devices [88,89]. For example, Therapy-WREX, based on the mobile arm support WREX, uses two four-bar linkages and elastic bands to passively counterbalance the weight of the arm, promoting performance of reaching and drawing movements through a wide workspace [88]. The assistance applied, measured as the amount of arm weight counterbalanced, can be selected by a clinician by adding or removing elastic bands, according to the impairment level exhibited by the participant. A similar approach has been developed for assisting in gait training, counterbalancing the weight of the leg using a gravity-balancing, passive exoskeleton [32]. Non-exoskeleton passive devices that reduce the amount of weight on the participant lower limbs have been developed to assist participants to train standing-balance [90], or to keep balance while walking overground [91].

It is also possible to actively generate a counterbalance force through the robot's control system to assist in reaching [18,92-94] or walking [14,29,95]. This active technique allows the selection of a weight support level via software to meet participants' individual needs, and can take into account other forces that can restrain participant's free movement such as those arising from abnormal tone [53,96] rather than just gravitational forces. For either passive or active counterbalance methods, the amount of weight support can be progressively reduced during training [16,88,92,94] to accommodate better for participant impairment level. We note that several recent devices provide at least some of the counterbalance mechanically for two practical reasons [17,33]: a power shutoff will not end in a free fall of the robot, and the effective force range of the actuators is extended.

### EMG-based assistance

Some groups have developed robotic devices that employ surface electromyography signals (sEMG) to drive the assistance. The EMG signals recorded from selected muscles (i.e pectoralis major, triceps, anterior middle and posterior deltoids, biceps, soleus, gastrocnemius), can be used as an indicator of effort generation to trigger assistance. An example of such an EMG triggered assistance was proposed with the MIT-MANUS robot [55], where EMG signals are collected from different muscles on the shoulder and elbow and, after some signal processing, the assistance is triggered when the processed EMG signals increase above a threshold. Similar approaches are proposed for upper limb rehabilitation in [19,25,87].

Other devices generate assisting forces proportional to the amplitude of the processed EMG in a kind of "proportional myoelectric control" for the arm [97-99], or for walking [30,100,101]. With this approach participants control their own movements, since they decide the movement to be performed, while the robotic device compensates for weakness, generating a force proportional to the EMG signal needed to drive the movement. There are some limitations in the use of EMG signals. For example, EMG signals are sensitive to electrode placement, interference from neighboring muscles signals, and skin properties (e.g. sweat on the skin, blood circulation), and dependent on the overall neurologic condition of the individual. Thus EMG parameters need to be calibrated for every individual and recalibrated for each experimental session. Another issue with this approach is that if the participant creates an abnormal, uncoordinated muscle activation pattern, the robot could move in an undesired way.

### Performance-based adaptation of task parameters

The assistive control algorithms reviewed to this point are static in the sense that they do not adapt controller parameters based on online measurement of the participant's performance. Adapting control parameters has the potential advantage that the assistance can be automatically tuned to the participant's individual changing needs, both throughout the movement and over the course of rehabilitation [10,55,102]. Adapting control parameters is a key part of "patient-cooperative training" strategies developed first for the Lokomat, in which the robot adaptively takes into account the patient's intention rather than imposing an inflexible control strategy [10]. It is also a key part of "performance-based, progressive robot-assisted therapy" control strategy developed for MIT-MANUS [55]. Several adaptive strategies have been proposed of the form:

(1)

where *P*_*i *_is the control parameter that is adapted (e.g. the movement timing, the gain of robot assistance force, or the robot stiffness), *i *refers to the *i*^*th *^movement, and *e*_*i *_is a performance error or measure, such as a measure of the participant's ability to initiate movement or ability to reach a target. This adaptive law is an error-based strategy that adjusts a control parameter from trial to trial based on measured participant performance. We denote the constants *f *and *g *as the forgetting and gain factors respectively. For MIT-MANUS, performance-based, progressive robot-assisted therapy used an algorithm like this with f = 1 [55]. A position-feedback type assisting controller was designed that allowed participants freedom to move more quickly than the desired trajectory (i.e. the virtual channel with a moving back wall). The duration of the desired trajectory and the stiffness of the robot controller were modified such that the reaching task was less demanding if the participant was more impaired. For the ARM Guide [102], a similar adaptive update law was proposed, with the performance variable being the maximum velocity during a reaching task, and the updated variable the coefficient of a "negative damping" term that helped drive the limb along the device. Other task parameters, such as the desired velocity [103], and desired movement time [7,104] have been adapted following similar adaptive laws. Such an algorithm was also altered to adjust impedance as follows:

(2)

where *G *represents the value of the robot impedance. When this algorithm was applied to the assisting robot's impedance at many samples of the step trajectory during walking, it was found to cause these impedances to converge to unique, low values that assisted the participants with SCI in stepping effectively [105]. This technique has also been used to reduce the assistance force provided during training of a driving task, promoting motor learning while limiting performance errors [46].

The inclusion of a forgetting term *f *in this sort of error-based adaptive controller is meant to address the possible problem of participant slacking in response to assistance. Without forgetting (*f *= 1), if the performance error is zero, the algorithm holds the control parameter constant, and the participant is not challenged further. However, if the forgetting factor is chosen such that 0 <*f *< 1, then the error-based learning algorithm reduces the control parameter when performance error is small, with the effect of always challenging the participant. Adaptive controllers with forgetting factors were recently proposed [50,94] in order to systematically reduce a feedforward assistive force for reaching when tracking errors are small. It is interesting to note that the human motor system itself apparently incorporates such a forgetting factor into an error-based learning law as it adapts to novel dynamic environments, in order minimize its own effort [6,106].

In the patient-cooperative framework, an adaptive impedance controller for the Lokomat was developed in which the machine impedance is increased when there is little participant effort detected, and decreased when participant effort is detected. An impedance-based adaptive control strategy has been proposed to control an ankle-foot orthosis to assist drop-foot gait in hemiparetic persons [107]. The robot's stiffness during controlled plantar flexion was adapted based on the number of foot slaps in the last 5 steps, thus reducing the stiffness by a fixed amount when no slaps were detected, or increasing the stiffness proportionally to the number of slaps when more than 2 slaps were detected.

Another approach to adaptive assistance is to use an optimization framework [10,108]. In the patient-cooperative framework, the robot attempts to minimize human-robot interaction torques in real-time [10]. Another approach is to pose the assistance-as-needed problem as a problem in which the goal is to minimize a cost that is the sum of kinematic error (ensuring the task is completed) and robotic assistance (ensuring that the robot assists as little as possible) [108]. This optimization problem was solved for the task of assisting unimpaired individuals in adapting to a perpendicular viscous force field applied to the leg during walking, resulting in an error-based assistive controller similar to the form of Equation 2 [108].

The need for adaptive controllers becomes more acute when the goal is to provide mechanically compliant assistance for movement. A stiff robot can simply drive the participant's limb(s) along a desired path. A compliant robot instead must calculate an appropriate amount of force to cancel the effects of increased tone, weakness, or lack of coordinated control by the participant. Tone, weakness, and lack of control vary widely between participants, suggesting use of adaptive or learning-based principles. In one study, an established adaptive control technique, a sliding-type, adaptive controller [16,50,109], was used to develop a radial-basis function model of the participant's force generation impairment, based on tracking error during a reaching task. When participants with stroke interacted with this controller, however, they allowed it to take over most of the work of lifting the arm (i.e. they slacked). A novel modification was thus made to the standard adaptive controller that made the robot attempt to reduce its force when tracking error is small, using a "forgetting" factor similar to those described above. Including this forgetting term encouraged more effort from the participants, preventing them from relying on the assistance, and also adapted the assistance to match the level of the participants' impairment. Interestingly, enhanced effort was achieved while allowing only a small increase in tracking error [16]. A similar adaptive algorithm has been proposed to learn a time-based model of forces for a reaching task [110].

## Determining the desired trajectory

Implementing assistance strategies, and indeed also many of the challenge strategies discussed in the next section, often requires a desired trajectory to be specified. The most common strategy for determining the desired trajectory is to model the trajectory based on normative movements (mathematical models of normative trajectories such as a minimum-jerk trajectory [18,20,24,34,50,55,56,60,80,103], pre-recorded trajectories from unimpaired volunteers [7,9,10,12,71,72,76,111], or pre-recorded trajectories during therapist-guided assistance [17,21,62,69,105]). We note, however, that there is no rigorous evidence that desired trajectories should be "normative" in order to maximally stimulate plasticity during motor training.

Another strategy for determining the desired trajectory, possible for bilateral tasks, is to base the desired trajectory on the movement of the "good" limb [45,53,54,57,58,75,111,112]. This approach was used with the MIME robotic rehabilitation device [45]. Motions of the unimpaired arm were detected and replicated by the robotic device that directed the movement of the affected limb based on position-control, thus facilitating bimanual movement practice. A similar approach was employed in the BiManuTrack device [58], for which the unimpaired extremity guided the affected limb in a mirror-like fashion, and for the LOPES gait training robot [111] where the state of the unimpaired leg was mapped, through a technique called Complementary Limb Motion Estimation, to determine the reference motion of the paretic limb. Bilateral strategies may even have a neurologic benefit: researchers have postulated benefits from training with bimanual movements related to the neurology of bilateral control in both, upper extremities [45,57,113], and lower extremities [75,112].

Adaptive approaches have also been used to adjust the desired trajectory. As mentioned above, one strategy adapted the desired trajectory based on contact forces between the robot and the limb [10]. Other strategies include re-planning the (minimum jerk) desired trajectory at every time sample based on the actual performance of the participant [114], or adjusting the replay-timing of the desired trajectory from time sample to time sample based on the difference between the actual, measured state of the participant and the desired state, with the effect of better synchronizing a compliant gait training robot to the participant [7].

The problem of determining the desired trajectory for a robotic therapy controller is essentially the problem of predicting human behavior for a given task – i.e. identifying a model of human motor behavior. For relatively simple tasks, such as point-to-point reaching, normative behavior is fairly well described (i.e. the. minimum jerk trajectory). Providing assistance for more complex tasks will require developing models of normative motor behavior for these tasks. For example, a recently developed controller predicts human steering motions during a driving task, allowing assistance to be provided in a beneficial way for this task [46].

Some robotic therapy controllers do not require desired trajectories. For example resistive strategies can be implemented without desired trajectories. EMG-proportional controllers do not require desired trajectories since the participant's self-selected EMG specifies the desired movement. Likewise, participants with counterbalanced limbs can participate freely in a wide variety of self-directed exercises. However, the ability to complete those exercises may be limited since a counterbalance approach may not restore full range of motion or coordination. For example, when the restraining forces due to neuromuscular tone and gravity were actively cancelled with a robot [96,115] or by chemically blocking antagonist muscle activity [116], persons post-stroke did not recover full range of motion of reaching or hand opening, respectively, suggesting that position-dependent agonist weakness substantially limits active range of motion. Thus, robotic devices that intend to aim movements across a large workspace need to account for position, velocity, tone, and gravity. Allowing a participant to use a brain-computer interface to specify a desired trajectory, or even robot forces, may allow greater participant control over the movement to be performed [117].

## Challenge-based robotic therapy control algorithms

Although the term "challenge-based controllers" is somewhat vague, we will use it to refer to controllers that in some ways make a task more difficult or challenging, as opposed to the assistive controllers reviewed above that make tasks easier in some way. This work on challenge-based controllers is providing insight that might be missed by focusing solely on assistive-type algorithms. As noted above, however, in some ways, challenge and assistive controllers can be viewed as being different points on the same continuum, a continuum along which task difficulty is modulated to optimally challenge the participant [118].

### Resistive strategies

Resistive exercise refers to the therapeutic strategy of providing resistance to the participant's hemiparetic limb movements during exercise, an approach that has a long history in clinical rehabilitation and clinical rehabilitation devices. For example, the "Proprioceptive Neurofacilitation (PNF)" therapy technique advocates for resisting participant's motions along "diagonal movement patterns" during rehabilitation training [119]. From one perspective, the first robotic therapy devices were computer-controller motors designed specifically for resistive training, such as the Biodex and Lido machines [120,121]. There is a reasonable amount of evidence now from multiple non-robotic studies that resistive type exercise that requires higher effort from the impaired limb can indeed help persons post-stroke improve motor function [122-126].

There have been a few attempts to incorporate resistive training into robotic therapy. Examples of resistive robotic devices that apply constant resistive forces to the affected limb, independent of its position or velocity, have been proposed for reaching and grasping practice [33,58-60,63,81], and walking [73,127]. Many of these robotic devices introduce resistance-based training just as one of the multiple therapy options of the robotic device, usually for participants with a low level of impairment. A more sophisticated resistance proposed in [45] consists of applying a viscous resistance consisting of a resistive force in the movement's direction proportional to the affected limb's velocity.

From one perspective, moving against gravity can be considered as a variant of the resistive approach, considering that gravity applies a force to the participant's limbs. Canceling gravity only as needed has been proposed with several robotic devices that can actively generate a counterbalance force through the robot's control system [9,50,92-94,128]. These devices have the ability to cancel only a percentage of the limb and robot weight, increasing the resistance on the participant's limb and demanding a higher effort from the impaired limb.

### Constraint-induced strategies

In the rehabilitation literature, the term "constraint-induced" therapy refers to a family of rehabilitation techniques in which the unimpaired limb of persons post-stroke is constrained (for example in a sling or with a mitt) to encourage use of the impaired limb [129]. Several robotic therapy control strategies have been developed consistent with the main idea of this strategy, which is to "force use" of the impaired limb.

For example, Johnson et al. [130] developed a robotic steering wheel that resists turning when the person post-stroke relies too heavily on his unimpaired arm, and showed that this approach encourages use of the impaired limb. Simon et al. [131] developed a robotic control strategy to improve force generation symmetry in the lower limbs, which applies resistance proportional to the difference between the force generated by both legs. For the "Guided Force Training" algorithm [96,102], subjects reach along a linear rail, and a robot halts the participant's movement if the participant pushes with an abnormally large force perpendicular to the rail. This strategy was inspired by the "active constrained" mode of MIME, which essentially only allowed the participant to move if force generation was toward the target [113].

### Error-amplification strategies

Assisting-type robotic therapy algorithms have the effect of reducing movement errors – they help the participant do the task better. However, research on motor adaption has emphasized that kinematic errors generated during movement are a fundamental neural signal that drives motor adaptation [6,132-134]. Thus, researchers have proposed robotic therapy algorithms that amplify movement errors rather than decrease them. Patton and colleagues [133,135] showed that amplifying curvature errors during reaching by persons with chronic stroke with a robotic force field caused participants to move straighter, at least temporarily, when the force field was removed, compared to reducing curvature errors during training. Similarly, Riesman et al. [134] increased limb phasing error in persons' post-stroke gait through a split-belt treadmill, thus increasing walking spatial-temporal asymmetries during a short adaptation session. The adaptation induced temporary after-effects causing walking symmetry in participants that showed asymmetries during baseline. Related work in this area showed that unimpaired subjects could be made to adapt more quickly by transiently amplifying their movement errors for the task of learning to walk in a robotic force field [132]. Several studies have shown that some benefits of error amplification can be achieved by distorting visual feedback from the task, rather than by physically altering movements [60,136,137].

## Haptic simulation strategies

Robotic therapy devices can be used as haptic interfaces for interacting with virtual reality simulations of activities of daily living, such as manipulating objects [17,18,21,63,64,138-144] or walking across a street [15,78,145,146]. Potential advantages of this approach over training in physical reality include: a haptic simulator can create many different interactive environments simulating a wide range of real-life situations, quickly switch between these environments without a "set-up" time, automatically grade the difficulty of the training environment by adding or removing virtual features, make the environments more interesting than a typical rehabilitation clinic (e.g. walking through Paris versus down a hospital hallway), automatically "reset" itself if objects are dropped or misplaced, and provide novel forms of visual and haptic feedback regarding performance. A variable of virtual environments was suggested by Lum et al. in [5], where real objects for manipulation where presented robotically. This technique resembles a robotic therapy system developed in 1989 [4] in which a robot arm was programmed to place physical targets for reaching and manipulation.

## Non-contacting coaches

A final area of development of robotic therapy control algorithms is for mobile robots that do not contact the participant but rather operate beside the participant, directing and encouraging therapy activities [3]. The question immediately arises as to whether a robot is necessary for this function, as a computer alone could give auditory and visual instructions and feedback. There is evidence however that people respond differently to "embodied" intelligence [147]. Therefore, physically embodying the coaching system in a robot may bring novel and relevant neuro-psychological mechanisms into play during movement training.

In this field, the development of the robot control algorithms focuses primarily on questions such as "How should the robot move and talk to encourage effort by the participant? " and "What type of exercises, and what practice order, should the robot specify to maximize learning? " The emergence of this field serves to highlight the key role that motivational factors and practice protocols play in rehabilitation therapy. A related field that is emerging in motor learning research and could be used to help design robot "coaches" for rehabilitation therapy is that of using computational models of learning to determine the best sequence of movements for maximizing adaptation to novel dynamic environments [148].

## Experimental evidence of effectiveness of various control strategies for robotic movement training

We close this survey of control strategies with a brief review of the experimental evidence of the effectiveness of the various control strategies for robotic therapy. For reviews of studies that have examined the effect of mechanical assistance on motor learning by unimpaired subjects, we refer the reader to [46,149].

Many studies of assistance techniques in robotic therapy have examined the effect of assistance given to chronic neurologic participants, with the participants' baseline motor status used as their own control. These studies show that robot assistance modestly but significantly decrease motor impairment, including at long-term follow-ups, using standard clinical scales as the outcome measures [56,58,59,77,83,88,98,113,135,150-156]. Other studies have shown that an additional dose of robot assistance relative to a group that received normal therapy improved recovery [157-160]. Impairment reduction with robotic therapy is often small enough to have marginal clinical or functional significance [161].

Perhaps more interesting for the purposes of this review are the few studies that have compared assistive control strategies to other controllers or rehabilitation strategies. For the upper extremity, one study compared impedance-based assistance to conventional therapy, and found a marginally significantly greater benefit [45]. A more recent study compared bilateral, unilateral, and combined bilateral and unilateral with conventional therapy and found that although combined unilateral and bilateral robotic training had advantages compared with conventional therapy, the differences did not hold in long term retention (6 month follow-up) [162]. A study that compared triggered assistance to no assistance and found no significant differences [79]. Training with impedance-based assistance compared to a smaller number of FES-triggered movements for wrist movements resulted in a significant and substantial advantage for the robot assistance strategy [57]. A comparison of an impedance-based assistance strategy to a resistance strategy for reaching after stroke found no significant difference [163]. Hogan et al. [86] compared performance-based progressive assistance to historical data from non-progressive assistance, and observed larger gains with the progressive assistance technique. A comparison of counterbalance assistance to traditional table top therapy found a small benefit with regards to impairment reduction, and revealed that participants strongly preferred the counterbalance assistance [164]. Similarly, a comparison of impedance-based robotic assistance to traditional sling suspension therapy found that the rate of recovery in the robotic group was greater than the sling suspension group for most subjects [165]. A recent study of a hand robot (HWARD) found that persons with chronic stroke who received a greater dose of time-triggered robotic assistance therapy applied using the robot experienced greater behavioral gains than a group of participants who received a smaller dose, plus active non-assist therapy (i.e. therapy in which the subjects did all the work and the robot does not assist) [157]. This may be the first direct evidence that robot assistance can be differentially beneficial as opposed to a matched amount of unassisted practice.

For gait training robots, there have been several recent studies that have compared impedance-based assistance to conventional rehabilitation techniques. One recent study found that intensive locomotor training from the electromechanical gait trainer GT I [166] plus physiotherapy resulted in a significantly better gait and basic activities of daily living ability in subacute stroke patients compared with conventional physiotherapy alone [158]. A study that compared impedance-based robotic assistance from the robot-driven gait orthosis Lokomat plus conventional therapy with conventional physiotherapy alone in hemiparetic patients after stroke found no significant difference between groups [159]. However, two recent studies [167,168] compared robot assistance from Lokomat to therapist-assisted conventional therapy for chronic stroke participants, and found substantially greater improvements in speed and single limb stance time from the conventional therapist-assisted locomotion training. One study in spinal-injured mice compared two forms of impedance control [71]. One strategy used a position-dependent, velocity force field with a deadband that assisted spinal-transected mice to step following a nominal step trajectory with bilateral coordination, and was compared with a fixed training trajectory, and an assist-as-needed strategy without enforced interlimb coordination. It was found that although all the training strategies increased stepping ability, the number of steps and periodicity (consistency of step timing) increased significantly more when the mice were trained with assistance-as-needed with interlimb coordination. The differential training effects were small, however.

Evidence of differential clinical benefits of training with challenge-based controllers is sparse. In what appears to be the only randomized controlled study of resistive versus assistive forces, Stein et al. [163] compared the motor outcomes of chronic stroke persons who exercised while receiving viscous resistance from MIT-MANUS, with a group that exercised while receiving impedance-based assistance. They found that both groups improved in various outcome measures, but that there were no significant differences between groups.

For robot control algorithm studies using a constraint-induced philosophy, a comparison study of the Guided Force Training algorithm with training free reaching and conventional occupational therapy found that persons post-stroke trained with the robotic device significantly increased upper extremity Fugl-Meyer scores, significantly decreased the time to perform the task and demonstrated a transfer of motor learning to functional tasks [169]. However robot training did not show greater gains when compared to the non-robotic strategies. A study with the MIME robotic system [113] provided some evidence of the effectiveness of the active-constrained mode robotic therapy reporting that directional force generation errors were reduced in six of eight movement patterns. Furthermore, low-level subjects increased their extent of reach, and high-level subjects increased their speed. The Driver's Seat approach increased effort from the unimpaired side [130].

Studies using training with error-amplification control strategies have shown short-term improvements in curvature during reaching [133,135] or interlimb coordination during walking, following chronic stroke. These improvements were not achieved with movement practice without error amplification. The long-term benefits of error-amplification (e.g. benefits of aftereffects) are unknown.

For haptic simulation techniques, there is some experimental evidence of effectiveness. One pilot study found that training with a haptic simulator/hand rehabilitator increased finger and thumb range of motion and/or speed in all 8 persons post-stroke [140]. Improvements showed during training in the virtual environment transferred to gains in functional real-world movements. Training in a virtual environment with a PHANToM™ haptic device increased participant's grip force generation, movement endurance and generated a more correct motor pattern [10]. Training in reaching and interacting with real objects did not show any detectable advantage over training with simulated objects with MIT-MANUS [170]. Training with a web-based virtual environment with a force feedback joystick improved movement ability in a person post-stroke, and was highly motivating [144]. Testing of a web-based haptic joystick rehabilitation suste, [171] and an ankle robot connected to a virtual reality simulator [172] resulted in high acceptance and satisfaction in a person post-stroke. Significantly, addition of virtual reality to robot-assisted lower extremity training was recently found to improve therapeutic outcomes, compared to robot-based training alone [173].

Finally, clinical testing with non-contact robotic coaches is still in an early stage. There are positive reports of participant compliance and satisfaction with the robot-specified exercises [3-5].

To summarize, while many studies have demonstrated that training with different robotic control strategies can significantly reduce motor impairment as assessed with standard clinical outcome measures, few studies have found differential benefits of particular robotic control strategies with respect to other robotic control strategies. Two recent studies [167,168] actually found that a particular form of robot assistance during gait training (relatively rigid, rote assistance) was substantially less effective than an equivalent dose of manual assistance from a physical therapist during the same motor task (walking on a treadmill). This negative finding highlights the important concept that the specific form of robot control selected for a rehabilitation application does indeed matter.

## Conclusion

We reviewed the development of robotic therapy control algorithms intended to promote neuroplasticity and motor learning during rehabilitation after neurologic injury. Even though a substantial amount of work has now been done, the field is rapidly evolving. The question of the most effective control algorithms is still wide open, in part because the randomized controlled trials necessary to identify these algorithms are expensive and time-consuming. Fundamentally, it is still even unclear whether robotic control approaches have the potential to produce greater benefits than is possible with simpler techniques, such as rote, unassisted practice [174]. We conclude by suggesting three directions for future research.

The first direction is to focus randomized controlled trials in this field on rigorous comparison of control algorithms with each other, and with simpler, non-robotic therapy approaches. There are now many studies that examined the effect of robotic therapy on chronic patients with the patients' baseline as their own control [56,58,59,77,83,88,98,113,135,150-156], and studies in which an additional dose of therapy in the form of robot therapy was given [157-160]. Giving the same dose of robotic therapy with two different control algorithms will help define which features of the control algorithms enhance motor recovery.

A second direction is to initiate use of more precision in defining what control algorithms are most appropriate for which rehabilitation tasks, what types of neurologic injuries, and at what stage of recovery. Motor learning and neuroplasticity mechanisms, and rehabilitation exercises themselves, are wonderfully diverse. Robotics has the opportunity to make a quantum leap by systematically implementing and controlling therapies, and by enabling systematic adjustment of treatment parameters. There is the beginning of a library of robotic therapy control algorithms from which to select, as reviewed here, to address these mechanisms and exercises. There are also experimental techniques that can precisely define features of neurologic injuries (e.g. medical imaging) and associated impairments (e.g. methods for quantifying weakness, tone, incoordination, and sensory deficits). Tailoring the control algorithm to the participant-specific pathophysiology, recovery stage, and the specific activity being rehabilitated may improve its therapeutic benefit. Mixtures and progressions of different robotic control strategies will likely end up being best; mixtures of robotics and FES or other training strategies are another possibility [76]. The form of feedback provided during robot-assisted training may be as important as the form of robot mechanical intervention itself [60,136,173].

The third direction is to develop better computational models of motor learning and recovery, in order to inform robot therapy control design. Developing such models may help in developing therapeutically better control algorithms using an optimization framework once the variables that drive adaptation are more clearly defined.

## Competing interests

The authors declare that they have no competing interests.

## Authors' contributions

LMC drafted the manuscript. DJR contributed concepts and edited and revised the manuscript. Both authors read and approved the manuscript.

## Supplementary Material

Additional file 1 **Summary of control strategies for robot-assisted therapy**. Table summarizing examples of control strategies for robot-assisted therapy for the upper and lower extremities. Click here for file

## References

[B1] Reinkensmeyer DJ, Emken JL, Cramer SC (2004). Robotics, motor learning, and neurologic recovery. Annual Review of Biomedical Engineering.

[B2] Riener R, Nef T, Colombo G (2005). Robot-aided neurorehabilitation of the upper extremities. Med Biol Eng Comput.

[B3] Matarić MJ, Eriksson J, Feil-Seifer DJ, Winstein CJ (2007). Socially assistive robotics for post-stroke rehabilitation. J Neuroeng Rehabil.

[B4] Kristy KA, Wu SJ, Erlandson RF, deBear P, Geer D, Dijkers M (1989). A robotic arm "smart exercise system": a rehabilitation therapy modality. Proceedings of the Annual International Conference of the IEEE Engineering in Medicine and Biology Society.

[B5] Lum PS, Uswatte G, Taub E, Hardin P, Mark VW (2006). A telerehabilitation approach to delivery of constraint-induced movement therapy. J Rehabil Res Dev.

[B6] Emken JL, Benitez R, Reinkensmeyer DJ (2007). Human-robot cooperative movement training: learning a novel sensory motor transformation during walking with robotic assistance-as-needed. J Neuroeng Rehabil.

[B7] Aoyagi D, Ichinose WE, Harkema SJ, Reinkensmeyer DJ, Bobrow JE (2007). A robot and control algorithm that can synchronously assist in naturalistic motion during body weight supported gait training following neurologic injury. IEEE Trans Neural Syst Rehabil Eng.

[B8] Veneman JF, Kruidhof R, Hekman EEG, Ekkelenkamp R, van Asseldonk EHF, Kooij H van der (2007). Design and evaluation of the LOPES exoskeleton robot for interactive gait rehabilitation. IEEE Trans Neural Syst Rehabil Eng.

[B9] Banala SK, Agrawal SK, Scholz JP (2007). Active Leg Exoskeleton (ALEX) for gait rehabilitation of motor-impaired patients. IEEE 10th International Conference on Rehabilitation Robotics, ICORR 2007.

[B10] Riener R, Lunenburger L, Jezernik S, Anderschitz JM, Colombo G, Dietz V (2005). Patient-cooperative strategies for robot-aided treadmill training: first experimental results. IEEE Trans Neural Syst Rehabil Eng.

[B11] Wheeler JW, Krebs HI, Hogan N (2004). An ankle robot for a modular gait rehabilitation system. Proceedings IEEE/RSJ International Conference on Intelligent Robots and Systems, IROS 2004.

[B12] Yano H, Kasai K, Saitou H, Iwata H (2003). Development of a gait rehabilitation system using a locomotion interface. The Journal of Visualization and Computer Animation.

[B13] Sawicki GS, Domingo A, Ferris DP (2006). The effects of powered ankle-foot orthoses on joint kinematics and muscle activation during walking in individuals with incomplete spinal cord injury. J Neuroeng Rehabil.

[B14] Surdilovic D, Zhang J, Bernhardt R (2007). STRING-MAN: Wire-robot technology for safe, flexible and human-friendly gait rehabilitation. IEEE 10th International Conference on Rehabilitation Robotics, ICORR 2007.

[B15] Schmidt H, Hesse S, Bernhardt R, Krüueger J (2005). HapticWalker-a novel haptic foot device. ACM Transactions on Applied Perception (TAP).

[B16] Wolbrecht ET, Chan V, Reinkensmeyer D, Bobrow JE (2008). Optimizing compliant, model-based robotic assistance to promote neurorehabilitation. IEEE Transactions on Neural Systems and Rehabilitation Engineering.

[B17] Nef T, Mihelj M, Riener R (2007). ARMin: a robot for patient-cooperative arm therapy. Medical and Biological Engineering and Computing.

[B18] Montagner A, Frisoli A, Borelli L, Procopio C, Bergamasco M, Carboncini MC, Rossi B (2007). A pilot clinical study on robotic assisted rehabilitation in VR with an arm exoskeleton device. Virtual Rehabilitation.

[B19] Perry JC, Rosen J, Burns S (2007). Upper-Limb powered exoskeleton design. IEEE/ASME Transactions on Mechatronics.

[B20] Wisneski KK, Johnson MJ (2007). Quantifying kinematics of purposeful movements to real, imagined, or absent functional objects: Implications for modelling trajectories for robot-assisted ADL tasks. Journal of NeuroEngineering and Rehabilitation.

[B21] Kousidou S, Tsagarakis NG, Smith C, Caldwell DG (2007). Task-orientated biofeedback system for the rehabilitation of the upper limb. IEEE 10th International Conference on Rehabilitation Robotics, 13–15 June ICORR.

[B22] Tsagarakism NG, Caldwell DG (2003). Development and control of a "soft-actuated" exoskeleton for use in physiotherapy and training. Autonomous Robots.

[B23] Zhang LQ, Park HS, Ren Y (2007). Developing an intelligent robotic arm for stroke rehabilitation. IEEE 10th International Conference on Rehabilitation Robotics, ICORR.

[B24] Loureiro RCV, Harwin WS (2007). Reach & grasp therapy: design and control of a 9-DOF robotic neuro-rehabilitation system. IEEE 10th International Conference on Rehabilitation Robotics, 13–15 June ICORR.

[B25] Krebs H, Volpe B, Williams D, Celestino J, Charles S, Lynch D, Hogan N (2007). Robot-aided neurorehabilitation: a robot for wrist rehabilitation. IEEE Transactions on Neural Systems and Rehabilitation Engineering.

[B26] Mayhew D, Bachrach B, Rymer WZ, Beer RF (2005). Development of the MACARM – a novel cable robot for upper limb neurorehabilitation. Proceedings of the 9th International Conference on Rehabilitation Robotics, ICORR.

[B27] Sugar TG, He J, Koeneman EJ, Koeneman JB, Herman R, Huang H, Schultz RS, Herring DE, Wanberg J, Balasubramanian S, Swenson P, Ward JA (2007). Design and control of RUPERT: A device for robotic upper extremity repetitive therapy. IEEE Transactions on Neural Systems and Rehabilitation Engineering.

[B28] Peshkin M, Brown DA, Santos-Munné JJ, Makhlin A, Lewis E, Colgate JE, Patton J, Schwandt D (2005). KineAssist: A robotic overground gait and balance training device. Proceedings of the 2005 IEEE 9th International Conference on Rehabilitation Robotics.

[B29] Steger R, Kim SH, Kazerooni H (2006). Control scheme and networked control architecture for the Berkeley lower extremity exoskeleton (BLEEX). Proceedings 2006 IEEE International Conference on Robotics and Automation, ICRA.

[B30] Hayashi T, Kawamoto H, Sankai Y (2005). Control method of robot suit HAL working as operator's muscle using biological and dynamical information. IEEE/RSJ International Conference on Intelligent Robots and Systems, IROS.

[B31] Miyoshi T, Hiramatsu K, Yamamoto SI, Nakazawa K, Akai M (2008). Robotic gait trainer in water: Development of an underwater gait-training orthosis. Disabil Rehabil.

[B32] Agrawal SK, Banala SK, Fattah A (2006). A gravity balancing passive exoskeleton for the human leg. Proceedings of Robotics: Science and Systems.

[B33] Stienen AHA, Hekman EEG, Helm FCT Van der, Prange GB, Jannink MJA, Aalsma AMM, Kooij H Van der (2007). Dampace: dynamic force-coordination trainer for the upper extremities. IEEE 10th International Conference on Rehabilitation Robotics, ICORR.

[B34] Rosati G, Gallina P, Masiero S (2007). Design, implementation and clinical tests of a wire-based robot for neurorehabilitation. IEEE Transactions on Neural Systems and Rehabilitation Engineering.

[B35] Vallery H, Ekkelenkamp R, Kooij H van der, Buss M (2007). Passive and accurate torque control of series elastic actuators. Proceedings of the IEEE/RSJ International Conference on Intelligent Robots and Systems, IROS.

[B36] Lotze M, Braun C, Birbaumer N, Anders S, Cohen LG (2003). Motor learning elicited by voluntary drive. Brain.

[B37] Perez MA, Lungholt BK, Nyborg K, Nielsen JB (2004). Motor skill training induces changes in the excitability of the leg cortical area in healthy humans. Exp Brain Res.

[B38] Reinkensmeyer DJ, Kahn LE, Averbuch M, McKenna-Cole AN, Schmit BD, Rymer WZ (2000). Understanding and treating arm movement impairment after chronic brain injury: Progress with the ARM Guides. J Rehabil Res Dev.

[B39] Hesse S, Kuhlmann H, Wilk J, Tomelleri C, Kirker S (2008). A new electromechanical trainer for sensorimotor rehabilitation of paralysed fingers: A case series in chronic and acute stroke patients. Journal of NeuroEngineering and Rehabilitation.

[B40] Poon CS (2004). Sensorimotor learning and information processing by Bayesian internal models. Proceedings of the 26th Annual International Conference of the IEEE Engineering in Medicine and Biology Society, IEMBS.

[B41] Rossini PM, Dal Forno G (2004). Integrated technology for evaluation of brain function and neural plasticity. Phys Med Rehabil Clin N Am.

[B42] Marchal-Crespo L, Reinkensmeyer DJ (2008). Effect of robotic guidance on motor learning of a timing task. Proceedings of the Second IEEE/RAS-EMBS International Conference on Biomedical Robotics and Biomechatronics.

[B43] Harkema SJ (2001). Neural plasticity after human spinal cord injury: application of locomotor training to the rehabilitation of walking. The Neuroscientist.

[B44] Reinkensmeyer DJ (2003). How to retrain movement after neurologic injury: a computational rationale for incorporating robot (or therapist) assistance. Proceedings of the 25th Annual International Conference of the IEEE Engineering in Medicine and Biology Society, IEMBS.

[B45] Lum PS, Burgar CG, Shor PC, Majmundar M, Loos M Van der (2002). Robot-assisted movement training compared with conventional therapy techniques for the rehabilitation of upper-limb motor function after stroke. Arch Phys Med Rehabil.

[B46] Marchal-Crespo L, Reinkensmeyer DJ (2008). Haptic guidance can enhance motor learning of a steering tasks. Journal of motor behaviour.

[B47] Reinkensmeyer DJ, Housman SJ (2007). "If I can't do it once, why do it a hundred times?": Connecting volition to movement success in a virtual environment motivates people to exercise the arm after stroke. Virtual Rehabilitation.

[B48] Schmidt RA, Bjork RA (1992). New conceptualizations of practice: common principles in three paradigms suggest new concepts for training. Psychological Science.

[B49] Israel JF, Campbell DD, Kahn JH, Hornby TG (2006). Metabolic costs and muscle activity patterns during robotic- and therapist-assisted treadmill walking in individuals with incomplete spinal cord injury. Physical Therapy.

[B50] Wolbrecht ET, Chan V, Le V, Cramer SC, Reinkensmeyer DJ, Bobrow JE (2007). Real-time computer modeling of weakness following stroke optimizes robotic assistance for movement therapy. 3rd International IEEE/EMBS Conference on Neural Engineering, CNE.

[B51] Krebs HI, Hogan N, Aisen ML, Volpe BT (1998). Robot-aided neurorehabilitation. Rehabilitation Engineering, IEEE Transactions on.

[B52] Aisen ML, Krebs HI, Hogan N, McDowell F, Volpe BT (1997). The effect of robot-assisted therapy and rehabilitative training on motor recovery following stroke. Archives of Neurology.

[B53] Lum PS, Reinkensmeyer DJ, Lehman SL (1993). Robotic assist devices for bimanual physical therapy: preliminary experiments. IEEE Transactions on Rehabilitation Engineering.

[B54] Lum PS, Lehman SL, Reinkensmeyer DJ (1995). The bimanual lifting rehabilitator: a device for rehabilitating bimanual control in stroke patients. IEEE Transactions on Rehabilitation Engineering.

[B55] Krebs HI, Palazzolo JJ, Dipietro L, Ferraro M, Krol J, Rannekleiv K, Volpe BT, Hogan N (2003). Rehabilitation robotics: performance-based progressive robot-assisted therapy. Autonomous Robots.

[B56] Amirabdollahian F, Loureiro R, Gradwell E, Collin C, Harwin W, Johnson G (2007). Multivariate analysis of the Fugl-Meyer outcome measures assessing the effectiveness of GENTLE/S robot-mediated stroke therapy. Journal of NeuroEngineering and Rehabilitation.

[B57] Hesse S, Werner C, Pohl M, Rueckriem S, Mehrholz J, Lingnau ML (2005). Computerized arm training improves the motor control of the severely affected arm after stroke: a single-blinded randomized trial in two centers. Stroke.

[B58] Hesse S, Schulte-Tigges G, Konrad M, Bardeleben A, Werner C (2003). Robot-assisted arm trainer for the passive and active practice of bilateral forearm and wrist movements in hemiparetic subjects. Arch Phys Med Rehabil.

[B59] Boian R, Sharma A, Han C, Merians A, Burdea G, Adamovich S, Recce M, Tremaine M, Poizner H (2002). Virtual reality-based post-stroke hand rehabilitation. Proceedings of Medicine Meets Virtual Reality.

[B60] Brewer BR, Klatzky R, Matsuoka Y (2006). Initial therapeutic results of visual feedback manipulation in robotic rehabilitation. International Workshop on Virtual Rehabilitation.

[B61] Denève A, Moughamir S, Afilal L, Zaytoon J (2008). Control system design of a 3-DOF upper limbs rehabilitation robot. Computer Methods and Programs in Biomedicine.

[B62] Toth A, Fazekas G, Arz G, Jurak M, Horvath M (2005). Passive robotic movement therapy of the spastic hemiparetic arm with REHAROB: report of the first clinical test and the follow-up system improvement. 9th International Conference on Rehabilitation Robotics, ICORR 2005.

[B63] Lambercy O, Dovat L, Gassert R, Burdet E, Teo CL, Milner T (2007). A Haptic Knob for rehabilitation of hand function. Neural Systems and Rehabilitation Engineering, IEEE Transactions on.

[B64] Masia L, Krebs HI, Cappa P, Hogan N (2007). Design and characterization of hand module for whole-arm rehabilitation following stroke. Mechatronics, IEEE/ASME Transactions on.

[B65] Jackson AE, Holt RJ, Culmer PR, Makower SG, Levesley MC, Richardson RC, Cozens JA, Williams MM, Bhakta BB (2007). Dual robot system for upper limb rehabilitation after stroke: the design process. Proceedings of the Institution of Mechanical Engineers, Part C: Journal of Mechanical Engineering Science.

[B66] Richardson R, Jackson A, Culmer P, Bhakta B, Levesley MC (2006). Pneumatic impedance control of a 3-d.o.f. physiotherapy robot. Advanced Robotics.

[B67] Fischer HC, Stubblefield K, Kline T, Luo X, Kenyon RV, Kamper DG (2007). Hand rehabilitation following stroke: A pilot study of assisted finger extension training in a virtual environment. Topics in Stroke Rehabilitation.

[B68] Frick EM, Alberts JL (2006). Combined use of repetitive task practice and an assistive robotic device in a patient with subacute stroke. Physical Therapy.

[B69] Mayr A, Kofler M, Saltuari L (2008). ARMOR: An electromechanical robot for upper limb training following stroke. A prospective randomised controlled pilot study. Handchirurgie Mikrochirurgie Plastische Chirurgie.

[B70] Rocon E, Belda-Lois JM, Ruiz AF, Manto M, Moreno JC, Pons JL (2007). Design and validation of a rehabilitation robotic exoskeleton for tremor assessment and suppression. IEEE Transactions on Neural Systems and Rehabilitation Engineering.

[B71] Cai LL, Fong AJ, Otoshi CK, Liang Y, Burdick JW, Roy RR, Edgerton VR (2006). Implications of assist-as-needed robotic step training after a complete spinal cord injury on intrinsic strategies of motor learning. Journal of Neuroscience.

[B72] Hesse S, Schmidt H, Werner C (2006). Machines to support motor rehabilitation after stroke: 10 years of experience in Berlin. J Rehabil Res Dev.

[B73] Yoon J, Ryu J, Lim K (2005). Reconfigurable ankle rehabilitation robot for various exercises. Journal of Robotic Systems.

[B74] Timoszyk WK, Nessler JA, Acosta C, Roy RR, Edgerton VR, Reinkensmeyer DJ, de Leon R (2005). Hindlimb loading determines stepping quantity and quality following spinal cord transection. Brain Research.

[B75] Kamnik R, Bajd T (2007). Does unilateral pedaling activate a rhythmic locomotor pattern in the nonpedaling leg in post-stroke hemiparesis?. J Neurophysiol.

[B76] Stauffer Y, Allemand Y, Bouri M, Fournier J, Clavel R, Metrailler P, Brodard R, Reynard F (2009). The WalkTrainer -A New Generation of Walking Reeducation Device Combining Orthoses and Muscle Stimulation. Neural Systems and Rehabilitation Engineering, IEEE Transactions on.

[B77] Colombo R, Pisano F, Micera S, Mazzone A, Delconte C, Carrozza M, Dario P, Minuco G (2005). Robotic techniques for upper limb evaluation and rehabilitation of stroke patients. IEEE Transactions on Neural Systems and Rehabilitation Engineering.

[B78] Ekkelenkamp R, Veltink P, Stramigioli S, Kooij H van der (2007). Evaluation of a Virtual Model Control for the selective support of gait functions using an exoskeleton. Proceedings of the IEEE 10th International Conference o nRehabilitation Robotics, ICORR.

[B79] Kahn LE, Zygman ML, Rymer WZ, Reinkensmeyer DJ (2006). Robot-assisted reaching exercise promotes arm movement recovery in chronic hemiparetic stroke: A randomized controlled pilot study. Journal of Neuroengineering and Rehabilitation.

[B80] Johnson MJ, Wisneski KJ, Anderson J, Nathan D, Smith RO (2006). Development of ADLER: The Activities of Daily Living Exercise Robot. The First IEEE/RAS-EMBS International Conference on Biomedical Robotics and Biomechatronics, BioRob.

[B81] Bi S, Ji L, Wang Z (2005). Robot-aided sensorimotor arm training methods based on neurological rehabilitation principles in stroke and brain injury patients. 27th Annual International Conference of the Engineering in Medicine and Biology Society, IEEE-EMBS.

[B82] Ju MS, Lin CC, Lin DH, Hwang IS, Chen SM (2005). A rehabilitation robot with force-position hybrid fuzzy controller: hybrid fuzzy control of rehabilitation robot. IEEE Transactions on Neural Systems and Rehabilitation Engineering.

[B83] Colombo R, Pisano F, Micera S, Mazzone A, Delconte C, Carrozza M, Dario P, Minuco G (2008). Assessing mechanisms of recovery during robot-aided neurorehabilitation of the upper limb. Neurorehabil Neural Repair.

[B84] Takahashi CD, Der-Yeghiaian L, Le VH, Cramer SC (2005). A robotic device for hand motor therapy after stroke. Proceedings of the 2005 IEEE International Conference on Rehabilitation Robotics.

[B85] Tung WL, Wu WL, Huang MH, Su FC, Chang mechanisms of recovery during robot-aided neurorehabilitation of the upper limb JJ (2007). Effects of robot-aided bilateral force-induced isokinetic arm training combined with conventional rehabilitation on arm motor function in patients with chronic stroke. Arch Phys Med Rehabil.

[B86] Hogan N, Krebs HI (2004). Interactive robots for neuro-rehabilitation. Restorative Neurology and Neuroscience.

[B87] Dipietro L, Ferraro M, Palazzolo JJ, Krebs HI, Volpe BT, Hogan N (2005). Customized interactive robotic treatment for stroke: EMG-triggered therapy. IEEE Transactions on Neural Systems and Rehabilitation Engineering.

[B88] Sanchez RJ, Liu J, Rao S, Shah P, Smith R, Cramer SC, Bobrow JE, Reinkensmeyer DJ (2006). Automating arm movement training following severe stroke: functional exercises with quantitative feedback in a gravity-reduced environment. IEEE Transactions on Neural and Rehabilitation Engineering.

[B89] Stienen AHA, Hekman EEG, Helm FCT Van der, Prange GB, Jannink MJA, Aalsma AMM, Kooij H Van der (2007). Freebal: dedicated gravity compensation for the upper extremities. IEEE 10th International Conference on Rehabilitation Robotics, ICORR.

[B90] Matjacic Z, Hesse S, Sinkjaer T (2003). BalanceReTrainer: A new standing-balance training apparatus and methods applied to a chronic hemiparetic subject with a neglect syndrome. NeuroRehabilitation.

[B91] Veg A, Popovic DB (2008). Walkaround: Mobile balance support for therapy of walking. IEEE Transactions on Neural Systems and Rehabilitation Engineering.

[B92] Sukal TM, Ellis MD, Dewald JPA (2006). Source of work area reduction following hemiparetic stroke and preliminary intervention using the ACT 3D system. IEEE Transactions on Neural Systems and Rehabilitation Engineering.

[B93] Jackson A, Culmer P, Makower S, Levesley M, Richardson R, Cozens A, Williams MM, Bhakta B (2007). Initial patient testing of iPAM – a robotic system for stroke rehabilitation. IEEE 10th International Conference on Rehabilitation Robotics, ICORR 2007.

[B94] Mihelj M, Nef T, Riener R (2007). A novel paradigm for patient-cooperative control of upper-limb rehabilitation robots. Advanced Robotics.

[B95] Frey M, Colombo G, Vaglio M, Bucher R, Jorg M, Riener R (2006). A novel mechatronic body weight support system. IEEE Transactions on Neural Systems and Rehabilitation Engineering.

[B96] Reinkensmeyer DJ, Takahashi CD, Timoszyk WK, Reinkensmeyer AN, Kahn LE (2000). Design of robot assistance for arm movement therapy following stroke, invited paper. Advanced Robotics.

[B97] Song R, Tong KY, Hu X, Li L (2008). Assistive control system using continuous myoelectric signal in robot-aided arm training for patients after stroke. IEEE Transactions on Neural Systems and Rehabilitation Engineering.

[B98] Stein J, Narendran K, McBean J, Krebs K, Hughes R (2007). Electromyography-controlled exoskeletal upper-limb-powered orthosis for exercise training after stroke. American Journal of Physical Medicine & Rehabilitation.

[B99] Li Q, Wang D, Du Z, Sun L (2005). A novel rehabilitation system for upper limbs. 27th Annual International Conference of the Engineering in Medicine and Biology Society, IEEE-EMBS.

[B100] Ferris DP, Czerniecki JM, Hannaford B (2005). An ankle-foot orthosis powered by artificial pneumatic muscles. Journal of Applied Biomechanics.

[B101] Kang SJ, Ryu JC, Ryu JW, Kim KH, Mun MS (2004). A real-time control of powered gait orthosis by bio signal. Proceedings of the 11th World Congress of the International Societyfor Prosthetics and Orthotics, Hong Kong.

[B102] Kahn LE, Rymer WZ, Reinkensmeyer DJ (2004). Adaptive assistance for guided force training in chronic stroke. Proceedings of the 26th Annual International Conference of the IEEE Engineering in Medicine and Biology Society Meeting, IEMBS.

[B103] Erol D, Sarkar N (2007). Intelligent control for robotic rehabilitation after stroke. Journal of Intelligent and Robotic Systems.

[B104] von Zitzewitz J, Bernhardt M, Riener R (2007). A novel method for automatic treadmill speed adaptation. IEEE Transactions on Neural Systems and Rehabilitation Engineering.

[B105] Emken JL, Harkema SJ, Beres-Jones J, Ferreira CK, Reinkensmeyer DJ (2008). Feasibility of manual teach-and-replay and continuous impedance shaping for robotic locomotor training following spinal cord injury. IEEE Transactions of Biomedical Engineering.

[B106] Reinkensmeyer DJ, Liu J, Emken JL, Bobrow JE (2004). The nervous system appears to minimize a weighted sum of kinematic error, force, and change in force when adapting to viscous environments during reaching and steppings. III Symp in Advances in Computational Motor Control.

[B107] Blaya JA, Herr H (2004). Adaptive control of a variable-impedance ankle-foot orthosis to assist drop-foot gait. IEEE Trans Neural Syst Rehabil Eng.

[B108] Emken JL, Bobrow JE, Reinkensmeyer DJ (2005). Robotic movement training as an optimization problem: Designing a controller that assists only as needed. IEEE 9th International Conference on Rehabilitation Robotics, ICORR.

[B109] Slotine JJE, Li W (1991). Applied nonlinear control.

[B110] Rosati G, Bobrow JE, Reinkensmeyer DJ (2008). Compliant control of post-stroke rehabilitation robots: using movement-specific models to improve controller performance. Proceedings of the ASME International Mechanical Engineering Congress & Exposition IMECE 2008.

[B111] Vallery H, van Asseldonk EHF, Buss M, Kooij H van der (2009). Reference Trajectory Generation for Rehabilitation Robots: Complementary Limb Motion Estimation.

[B112] Kautz SA, Patten C (2005). Interlimb influences on paretic leg function in poststroke hemiparesis. Journal of Neurophysiology.

[B113] Lum PS, Burgar CG, Shor PC (2004). Evidence for improved muscle activation patterns after retraining of reaching movements with the MIME robotic system in subjects with post-stroke hemiparesis. IEEE Transactions on Neural Systems and Rehabilitation Engineering.

[B114] Wolbrecht E (2007). Adaptive, assist-as-needed control of a pneumatic orthosis for optimizing robotic movement therapy following stroke. PhD thesis.

[B115] Erol D, Sarkar N (2007). Smooth human-robot interaction in robot-assisted rehabilitation. IEEE 10th International Conference on Rehabilitation Robotics, ICORR.

[B116] Kamper DG, Harvey RL, Suresh S, Rymer WZ (2003). Relative contributions of neural mechanisms versus muscle mechanics in promoting finger extension deficits following stroke. Muscle Nerve.

[B117] Daly J, Wolpaw J (2008). Brain-computer interfaces in neurological rehabilitation. Lancet Neurol.

[B118] Guadagnoli M, Lee T (2004). Challenge point: a framework for conceptualizing the effects of various practice conditions in motor learning. J Mot Behav.

[B119] Voss DE, Ionta MK, Meyers BJ (1985). Proprioceptive Neurofacilitation: Patterns & Techniques.

[B120] Patterson LA, Spivey WE (1992). Validity and reliability of the LIDO active isokinetic system. Journal of Orthopaedic Sports Physical Therapy.

[B121] Feiring DC, Ellenbecker TS, Dersheid GL (1990). Test-retest reliability of the Biodex isokinetic dynamometer. Journal of Orthopaedic Sports Physical Therapy.

[B122] Weiss A, Suzuki T, Bean J, Fielding RA (2000). High intensity strength training improves strength and functional performance after stroke. Am J Phys Med Rehabil.

[B123] Ouellette MM, LeBrasseur NK, Bean JF, Phillips E, Stein J, Frontera WR, Fielding RA (2004). High-intensity resistance training improves muscle strength, self-reported function, and disability in long-term stroke survivors. Stroke.

[B124] Morris SL, Dodd KJ, Morris ME (2004). Outcomes of progressive resistance strength training following stroke: a systematic review. Clinical Rehabilitation.

[B125] Patten C, Dozono J, Schmidt S, Jue M, Lum P (2006). Combined functional task practice and dynamic high intensity resistance training promotes recovery of upper-extremity motor function in post-stroke hemiparesis: a case study. Journal of Neurologic Physical Therapy.

[B126] Mercier C, Bourbonnais D, Bilodeau S, Lemay JF, Cross P (1999). Description of a new motor re-education programme for the paretic lower limb aimed at improving the mobility of stroke patients. Clinical Rehabilitation.

[B127] Lam T, Wirz M, Lüunenburger L, Dietz V (2008). Swing phase resistance enhances flexor muscle activity during treadmill locomotion in incomplete spinal cord injury. Neurorehabil Neural Repair.

[B128] Ellis MD, Sukal T, DeMott T, Dewald JPA (2008). Augmenting Clinical Evaluation of Hemiparetic Arm Movement With a Laboratory-Based Quantitative Measurement of Kinematics as a Function of Limb Loading. Neurorehabil Neural Repair.

[B129] Shaw SE, Morris DM, Uswatte G, McKay S, Meythaler JM, Taub E (2005). Constraint-induced movement therapy for recovery of upper-limb function following traumatic brain injury. J Rehabil Res Dev.

[B130] Johnson MJ, Loos HFM Van der, Burgar CG, Shor P, Leifer LJ (2003). Design and evaluation of Driver's SEAT: A car steering simulation environment for upper limb stroke therapy. Robotica.

[B131] Simon AM, Gillespie RB, Ferris DP (2007). Symmetry-based resistance as a novel means of lower limb rehabilitation. Journal of Biomechanics.

[B132] Emken JL, Reinkensmeyer DJ (2005). Robot-Enhanced motor learning: accelerating internal model formation during locomotion by transient dynamic amplification. IEEE Transactions on Neural Systems and Rehabilitation Engineering.

[B133] Patton JL, Stoykov ME, Kovic M, Mussa-Ivaldi FA (2006). Evaluation of robotic training forces that either enhance or reduce error in chronic hemiparetic stroke survivors. Experimental Brain Research.

[B134] Reisman DS, Wityk R, Silver K, Bastian AJ (2007). Locomotor adaptation on a split-belt treadmill can improve walking symmetry post-stroke. Brain.

[B135] Patton JL, Kovic M, Mussa-Ivaldi FA (2006). Custom-designed haptic training for restoring reaching ability to individuals with poststroke hemiparesis. J Rehabil Res Dev.

[B136] Wei Y, Bajaj P, Scheidt R, Patton J (2005). Visual error augmentation for enhancing motor learning and rehabilitative relearning. 9th International Conference on Rehabilitation Robotics, ICORR.

[B137] Brewer BR, Klatzky R, Matsuoka Y (2008). Visual feedback distortion in a robotic environment for hand rehabilitation. Brain Research Bulletin.

[B138] Patton JL, Dawe G, Scharver C, Mussa-Ivaldi FA, Kenyon R (2004). Robotics and virtual reality: the development of a life-sized 3-D system for the rehabilitation of motor function. 26th Annual International Conference of the IEEE Engineering in Medicine and Biology Society, IEMBS.

[B139] Burdea GC (2003). Virtual rehabilitation-benefits and challenges. Methods of Information in Medicine.

[B140] Adamovich SV, Merians AS, Boian R, Tremaine M, Burdea GS, Recce M, Poizner H (2004). A virtual reality based exercise system for hand rehabilitation post-stroke: transfer to function. 26th Annual International Conference of the IEEE Engineering in Medicine and Biology Society, IEMBS.

[B141] Broeren J, Georgsson M, Rydmark M, Sunnerhagen KS (2002). Virtual reality in stroke rehabilitation with the assistance of haptics and telemedicine. Proceedings of the 4th International Conference on Disability, Virtual Reality and Associated Technologies.

[B142] McLaughlin M, Rizzo A, Jung Y, Peng W, Yeh SC, Zhu W (2005). Haptics-enhanced virtual environments for stroke rehabilitation. Procedings on IPSI 2005.

[B143] Carignan C, Liszka M, Roderick S (2005). Design of an arm exoskeleton with scapula motion for shoulder rehabilitation. Proceedings on the 12th International Conference on Advanced Robotics, ICAR.

[B144] Reinkensmeyer D, Pang C, Nessler J, Painter C (2002). Web-based telerehabilitation for the upper extremity after stroke. IEEE Transactions on Neural Systems and Rehabilitation Engineering.

[B145] Fung J, Malouin F, McFadyen BJ, Comeau F, Lamontagne A, Chapdelaine S, Beaudoin C, Laurendeau D, Hughey L, Richards CL (2004). Locomotor rehabilitation in a complex virtual environment. Proceedings of the 26th Annual International Conference of the IEEE Engineering in Medicine and Biology Society, IEMBS.

[B146] Boian RF, Deutsch JE, Su Lee C, Burdea GC, Lewis J (2003). Haptic effects for virtual reality-based post-stroke rehabilitation. Proceedings on the 11th Symposium on Haptic Interfaces for Virtual Environment and Teleoperator Systems, HAPTICS.

[B147] Reeves B, Nass C (1998). The Media Equation: How People Treat Computers, Television, and New Media Like Real People and Places.

[B148] Huang VS, Shadmehr R, Diedrichsen J (2008). Active learning: learning a motor skill without a coach. Journal of Neurophysiology.

[B149] Reinkensmeyer DJ, Patton JL (2009). Can robots help the learning of skilled actions?. Exercise and Sports Sciences Reviews.

[B150] Prange GB, Jannink MJ, Groothuis-Oudshoorn CG, Hermens HJ, Ijzerman MJ (2006). Systematic review of the effect of robot-aided therapy on recovery of the hemiparetic arm after stroke. J Rehabil Res Dev.

[B151] Ferraro M, Palazzolo JJ, Krol J, Krebs HI, Hogan N, Volpek BT (2003). Robot-aided sensorimotor arm training improves outcome in patients with chronic stroke. Neurology.

[B152] Meyer-Heim A, Borggraefe I, Ammann-Reiffer C, Berweck S, Sennhauser FH, Colombo G, Knecht B, Heinen F (2007). Feasibility of robotic-assisted locomotor training in children with central gait impairment. Dev Med Child Neurol.

[B153] Wirz M, Zemon DH, Rupp R, Scheel A, Colombo G, Dietz V, Hornby TG (2005). Effectiveness of automated locomotor training in patients with chronic incomplete spinal cord injury: a multicenter trial. Arch Phys Med Rehabil.

[B154] Macclellan LR, Bradham DD, Whitall J, Volpe B, Wilson PD, Ohlhoff J, Meister C, Hogan N, Krebs HI, Bever CTJ (2005). Robotic upper-limb neurorehabilitation in chronic stroke patients. J Rehabil Res Dev.

[B155] Finley MA, Fasoli SE, Dipietro L, Ohlhoff J, Macclellan L, Meister C, Withall J, Macko R, Bever C, Krebs HI, Hogan N (2005). Short-duration robotic therapy in stroke patients with severe upper-limb motor impairment. J Rehabil Res Dev.

[B156] Krebs H, Dipietro L, Levy-Tzedek S, Fasoli S, Rykman-Berland A, Zipse J, Fawcett J, Stein J, Poizner H, Lo A, Volpe B, Hogan N (2008). A paradigm shift for rehabilitation robotics. IEEE Engineering in Medicine and Biology Magazine.

[B157] Takahashi CD, Der-Yeghiaian L, Vu L, Motiwala RR, Cramer SC (2008). Robot-based hand motor therapy after stroke. Brain.

[B158] Pohl M, Werner C, Holzgraefe M, Kroczek G, Mehrholz J, Wingendorf I, Hooelig G, Koch R, Hesse S (2007). Repetitive locomotor training and physiotherapy improve walking and basic activities of daily living after stroke: a single-blind, randomized multicentre trial (DEutsche GAngtrainerStudie, DEGAS). Clinical Rehabilitation.

[B159] Husemann B, Mueller F, Krewer C, Heller S, Koenig E (2007). Effects of locomotion training with assistance of a robot-driven gait orthosis in hemiparetic patients after stroke: a randomized controlled pilot study. Stroke.

[B160] Mayr A, Kofler M, Quirbach E, Matzak H, Frohlich K, Saltuari L (2007). Prospective, blinded, randomized crossover study of gait rehabilitation in stroke patients using the lokomat gait orthosis. Neurorehabil Neural Repair.

[B161] Kwakkel G, Kollen BJ, Krebs HI (2008). Effects of Robot-Assisted Therapy on Upper Limb Recovery After Stroke: A Systematic Review. Neurorehabil Neural Repair.

[B162] Lum PS, Burgar CG, Loos M Van der, Shor PC, Majmundar M, Yap R (2006). Links MIME robotic device for upper-limb neurorehabilitation in subacute stroke subjects: A follow-up study. J Rehabil Res Dev.

[B163] Stein J, Krebs HI, Frontera WR, Fasoli SE, Hughes R, Hogan N (2004). Comparison of two techniques of robot-aided upper limb exercise training after stroke. Am J Phys Med Rehabil.

[B164] Housman SJ, Scott K, Reinkensmeyer DJ (2009). A Randomized Controlled Trial of Gravity-Supported, Computer-Enhanced Arm Exercise for Individuals With Severe Hemiparesis. Neurorehabilitation Neural Repair.

[B165] Coote S, Murphy B, Harwin W, Stokes E (2008). The effect of the GENTLE/s robot-mediated therapy system on arm function after stroke. Clinical Rehabilitation.

[B166] Hesse S, Werner C, Uhlenbrock D, Frankenberg SV, Bardeleben A, Brandl-Hesse B (2001). An electromechanical gait trainer for restoration of gait in hemiparetic stroke patients: Preliminary results. Neurorehabil Neural Repair.

[B167] Hornby TG, Campbell DD, Kahn JH, Demott T, Moore JL, Roth HR (2008). Enhanced gait-related improvements after therapist- versus robotic-assisted locomotor training in subjects with chronic stroke: a randomized controlled study. Stroke.

[B168] Hidler J, Nichols D, Pelliccio M, Brady K, Campbell D, Kahn J, Hornby T (2009). Multicenter randomized clinical trial evaluating the effectiveness of the Lokomat in subacute stroke. Neurorehabil Neural Repair.

[B169] Fischer H, Kahn L, Pelosin E, Roth H, Barbas J, Rymer W, Reinkensmeyer D (2006). Can Robot-Assisted Therapy Promote Generalization of Motor Learning Following Stroke?: Preliminary Results. The First IEEE/RAS-EMBS International Conference on Biomedical Robotics and Biomechatronics, BioRob.

[B170] Krebs HI, Mernoff S, Fasoli SE, Hughes R, Stein J, Hogan N (2008). A comparison of functional and impairment-based robotic training in severe to moderate chronic stroke: a pilot study. NeuroRehabilitation.

[B171] Sugarman H, Dayan E, Lauden A, Weisel-Eichler A, Tiran J (2008). Investigating the use of force feedback joysticks for low-cost, robot-mediated therapy. International Journal on Disability and Human Development.

[B172] Deutsch JA, Lewis JA, Whitworth E, Boian R, Burdea G, Tremaine M (2005). Formative evaluation and preliminary findings of a virtual reality telerehabilitation system for the lower extremity. Presence: Teleoperators and Virtual Environments.

[B173] Mirelman A, Bonato P, Deutsch J (2009). Effects of training with a robot-virtual reality system compared with a robot alone on the gait of individuals after stroke. Stroke.

[B174] Kahn LE, Lum PS, Rymer WZ, Reinkensmeyer DJ (2006). Robot-assisted movement training for the stroke-impaired arm: Does it matter what the robot does?. J Rehabil Res Dev.

