# Supplementary material for: Review of control strategies for robotic movement training after neurologic injury
Source: J Neuroeng Rehabil. 2009 Jun 16;6:20. doi: 10.1186/1743-0003-6-20 (PMC2710333; doi:10.1186/1743-0003-6-20)
Supplement: Additional file 1 — Summary of control strategies for robot-assisted therapy. Table summarizing examples of control strategies for robot-assisted therapy for the upper and lower extremities. [file 1743-0003-6-20-S1.pdf]

Control strategies for robot-assisted therapy			Examples for upper extremity	Examples for lower extremity
**Assistance Strategies**		Position control	[17,18,20–22,24,25, 34,45,51–70]	[8–12,15,28,31, 71–75,111,112]
• Make task safer to allow practice<br>• Enhance somatosensory input in a way correlated with motor output<br>• Increase task success to motivate practice<br>• Provide appropriate challenge point for optimal learning<br>• Reinforce normative sensory-motor pathways<br>• Interleave effort, which stimulates neuroplasticity, with stretching, which reduces soft tissue and reflex stiffness<br>• Allow more repetitions, since participant completes movements more efficiently	Impedance-based	channel	[17, 18, 56, 80–82]	[9]
		Velocity-field channel	[71]	[77]
		Moving back wall	[45, 50, 55]	[71]
		Time/force/error-based trigger	[24,27,38,45,55,56, 77, 79, 83–86]	[9]
	Counterbalance-based	Passive gravity counterbalancing	[17, 33, 88, 89]	[32, 90, 91]
		Active weight compensation	[18, 92–94]	[14, 29, 95]
		Counterpoise control	[23, 96]	...
	EMG-based	EMG-triggered impedance	[19, 25, 55, 87]	...
		force proportional to EMG	[97–99]	[30, 100, 101]
	Performance-based adaptive assistance	Modulate stiffness	[55, 94]	[10, 105, 107]
		Modulate desired movement time	[55]	[7, 103, 104]
		Modulate desired movement path	[50]	[10]
		Modulate assistance force	[46]	...
		Learn static model of weakness	[50]	...
		Learn time-based model of forces	[110]	...
		Adjust unstable force field gain	[102]	...
		Minimize sum of error and effort	...	[6]
**Challenge-Based Strategies**	Resistance	Constant resistance	[33, 58–60, 81, 108, 123–126, 163]	[73, 120, 121]
• Increase neural and muscle plasticity by increasing activation and force<br>• Discourage abnormal movements or disuse of impaired limb<br>• Drive learning with larger errors		Viscous resistance	[45]	[127]
		Cancel gravity only as needed	[50, 92–94, 128]	[9]
	Constraint-induced	Resist asymmetric movements	...	[131]
		Resist movement of unimpaired arm	[130]	...
		Halt movement if off-axis forces are large	[96, 102, 113]	...
	Error-amplification	Increase kinematic error	[133]	[132]
		Amplify visual representation of error	[135–137]	...
		Increase limb phasing error	...	[134]
**Haptic Simulation Strategies**<br>Make practice more flexible, convenient, safe, and motivating	Simulate interaction with physical objects		[17, 18, 21, 63, 64, 138–144]	[15, 78, 145, 146, 172]
	Robotically present real objects for manipulation		[4, 5]	...
**Embodied Coaching Strategies**<br>Motivate practice with an embodied coach	Mobile robot gives instructions based on monitored movement		[3]	
